# Associations of trimethylamine N-oxide (TMAO) and its precursors with childhood obesity: a case-control study

**DOI:** 10.1186/s12902-025-02075-z

**Published:** 2025-11-25

**Authors:** Yao Li, Xiancheng Wang, Manting Chen, Meiqun Xiao, Liya Ma, Tao Zhou, Mengying Wang, Qiying Song

**Affiliations:** 1 Department of Obstetrics, Shenzhen Baoan Women’s and Children’s Hospital, Shenzhen, 518100 China; 2 Department of Pediatrics, Shenzhen Hospital, Southern Medical University, Shenzhen, 518100 China; 3 Department of Child Healthcare, Shenzhen Baoan Women’s and Children’s Hospital, Shenzhen, 518100 China; 4 Department of Epidemiology and Biostatistics, School of Public Health (Shenzhen), Shenzhen Campus of Sun Yat-sen University, Shenzhen, 518107 China; 5 Department of Nutrition and Food Hygiene, School of Public Health, Peking University, Beijing, 100191 China; 6 Ministry of Education, Key Laboratory of Epidemiology of Major Diseases (Peking University), Beijing, 100191 China; 7 Health Science Center, Shenzhen University, Shenzhen, 518055 China

**Keywords:** Children, Obesity, Trimethylamine N-oxide (TMAO), Choline, Carnitine, Gut microbiota-derived metabolites

## Abstract

**Background:**

Emerging studies suggests a potential link between serum trimethylamine N-oxide (TMAO) and obesity in adults. However, data on this association in children remain scarce. The present study aimed to investigate the association of TMAO and its precursors with childhood obesity and to evaluate whether such associations would be modified by basic clinical characteristics.

**Methods:**

We conducted a case-control study involving 50 children with obesity and 50 children with normal weight aged 2–6 years. Serum TMAO and its precursors were quantified by ultra high-performance liquid chromatography-tandem mass spectrometry (UHPLC-MS/MS). Multivariate logistic regression models and Pearson correlation analyses were performed after controlling several confounding factors.

**Results:**

Serum choline was inversely associated with childhood obesity (OR (95% CI) for highest vs. lowest quartile: 0.16 (0.04, 0.61), *p =* 0.007), whereas serum creatinine and carnitine showed positive correlations (ORs: 30.03 and 5.31, *p* < 0.001 and *p* = 0.011 respectively). We also found that choline was negatively correlated with BMI (*p* = 0.038), whereas creatinine and carnitine were positively correlated with BMI (*p* < 0.001 and *p* = 0.002, respectively). in addition, subgroup analyses based on sex, age groups, and maternal gestational diabetes mellitus status showed generally consistent results, with no significant interactions. No significant associations of betaine, TMA and TMAO with childhood obesity were observed (all *p* > 0.05).

**Conclusion:**

Higher serum choline levels were associated with lower odds of childhood obesity, while elevated creatinine and carnitine levels were associated with higher odds. These findings provide hints regarding the potential significance of these metabolites, which can be influenced by gut microbiota activity, diet, and host metabolism, in early-life obesity.

**Clinical trial number:**

Not applicable.

**Supplementary Information:**

The online version contains supplementary material available at 10.1186/s12902-025-02075-z.

## Introduction

Childhood obesity is a major global public health concern requiring urgent attention. Over the past few decades, the prevalence of childhood obesity has witnessed a remarkable escalation worldwide, from 1.7% in 1990 to 6.9% in 2022 in girls and from 2.1% to 9.3% in boys during the same period [1]. According to the World Health Organization (WHO), 37 million children under the age of 5 were living with excess weight (overweight + obesity) globally in 2022, and over 390 million children and adolescents aged 5–19 years had excess weight, among whom 160 million were obese [2]. This issue is particularly critical in China, where without effective control, the prevalence of childhood obesity is projected to surge to 15.1% by 2030 due to rapid economic development and lifestyle transition, placing a substantial burden on the Chinese healthcare system [3].

Obesity in early life can lead to adverse short-term outcomes—such as impaired physical, mental, and cognitive development—and persist into adulthood, elevating the risk of chronic diseases including hypertension, diabetes mellitus, and cardiovascular conditions [3–6]. Clarifying the mechanisms underlying childhood obesity is essential for developing effective prevention and intervention strategies.

Recently, emerging studies have related gut microbiota-derived metabolites, such as trimethylamine N-oxide (TMAO) and its precursors (including betaine, choline, creatinine, carnitine, and TMA) with obesity [7–10]. Dehghan et al. [8] conducted a meta-analysis of 12 observational studies involving 17628 adults, and revealed a positive dose-dependent association between circulating TMAO concentration and obesity. Mihuta et al. [7] found in children aged 4 to 18 that significantly elevated levels of TMAO were observed in children with obesity compared to those with normal weight. Another study from Spain indicated that lifestyle intervention decreases urine TMAO levels in children with obesity [11]. Possible underlying mechanisms might involve the role of TMAO-producing enzyme (i.e., flavin-containing monooxygenase-3 (FMO3)) in regulating obesity and beiging of white adipose tissue [12], as well as increased hepatic insulin resistance correlate with elevated TMAO concentration [13]. Although a growing number of adult-based studies suggest that elevated TMAO levels may increase the risk of obesity, data specific to children remain scarce. Moreover, children harbor a distinct gut microbiome from adults [14, 15], which might potentially alter the relationship between gut microbiota-derived metabolites and obesity. Consequently, it is worthwhile to conduct further investigations in the pediatric population.

In the current study, we aimed to explore whether serum TMAO and its precursors are associated with obesity in young children (2~6 years old) in China. In addition, sex and age are fundamental demographic factors known to influence childhood obesity. Furthermore, there is growing evidence that intrauterine exposure to gestational diabetes mellitus (GDM) programs the offspring’s long-term metabolic health, predisposing them to adiposity and altered energy metabolism [16, 17]. Therefore, we also examined whether the associations between the above-mentioned serum metabolites and obesity varied by sex, age, and maternal GDM status.

## Methods

### Study participants

We conducted a case-control study comprising 100 Han Chinese children (50 children with obesity and 50 children with normal weight) aged 2 to 6 years recruited from five kindergartens in Baoan District of Shenzhen, China in 2024. All children with obesity in the selected kindergartens were included with their voluntary participation and the consent of their parents. Controls were chosen from the remaining children who were normal-weight in the same classes of the same kindergartens.

The study was approved by the Ethical Committees of Shenzhen Baoan Women’s and Children’s Hospital (approval number: LLSC 2021–04-01–02-KS). Written informed consent was obtained from all participants and their parents, and all methods were carried out in accordance with Declaration of Helsinki.

## Body measurement

The weight and height of children were measured wearing light clothing, bareheaded, and barefoot with a standard measuring stations and column scales (Seca 799 s, Seca, Hamburg, Germany). All measurements were conducted by trained nurses and each measurement was performed twice and the average was used for analysis.

Body mass index (BMI) was calculated as weight (kg) divided by the squared height (m). Based on the Growth standard for children under 7 years of age (WS/T 423–2022, available at https://www.nhc.gov.cn/wjw/c100311/wsbz.shtml) [18], which was released by the National Health Commission of the People’s Republic of China, the participants were defined as obese (age- and gender-specific BMI ≥ Median + 2 standard deviation (SD)) or normal-weight (Median − 2 SD ≤ age- and gender-specific BMI < Median + 1 SD).

In addition, we also reclassified the nutritional status of children in our study using the WHO Child Growth Standards. For children under 5 years of age, obesity are weight-for-height > 3 SD above the WHO Child Growth Standards median; for children aged between 5 and 6 years, obesity are BMI-for-age > 2 SD above the WHO Growth Reference median [2].

## Detection of serum TMAO and its precursors

Blood samples were collected after an overnight fast of at least 8 h. Serum samples were extracted and stored in a − 80 °C freezer within 24 hours until detection. Serum concentrations of TMAO-related metabolites (i.e., betaine, choline, creatinine, free carnitine, TMA, and TMAO in the present study) were determined by the stable isotope dilution ultra high performance liquid chromatography coupled tandem mass spectrometry (UHPLC-MS/MS) method (QTRAP 5500, AB Sciex, MA, USA). Briefly, a volume of 50 μL of the sample, 148 μL of the precipitating agent (methanol : acetonitrile : water = 4 : 4 : 2) and 2 μL of the mixed internal standard solution (250 μg/mL d9-betaine, 125 μg/mL d4-choline, 250 μg/mL d3-creatinine, 0.4 μg/mL d3-carnitine, and 250 μg/mL d9-TMAO; external standard was applied for TMA) were combined, vortex-mixed for 2 minutes, and then centrifuged at 15,000 rpm and 4 °C for 15 minutes. Subsequently, a volume of 1 μL of the supernatant was analyzed after injection into a ACQULTY UPLC BEH HILIC column (2.1 mm × 100 mm, 1.7 μm) and equilibrated with 20% solution A (10 mmol/L ammonium formate and 0.1% formate acid in water) and 80% solution B (acetonitrile) under isocratic elution with the flow rate of 0.4 mL/min. More details of the calibration and quality control, UHPLC-MS/MS parameters, and analytical methodology validation were provided in the **Supplementary Methods**.

## Statistical analysis

Continuous characteristics were analyzed using unpaired t-tests and reported as means ± SD and *M* (*P*_25_, *P*_75_). Categorical variables were compared by the chi-square test and presented as numbers and percentage. TMAO and its precursors were categorized into four quintiles, and multivariate logistic regression models were applied to estimate the odds ratios (ORs) and 95% confidence intervals (CIs) of TMAO and its precursors in relation to childhood obesity, with the lowest quintile (i.e., quintile 1) as the reference group. Both the unadjusted model and the adjusted model with gender, age, and maternal GDM status were included. Pearson correlation analysis were conducted to evaluate the rude correlations between serum TMAO or its precursors levels with BMI. We also performed subgroup analyses stratified by basic clinical characteristics, including gender, age groups, and maternal GDM status, and evaluate the interactions between these factors and TMAO-related metabolites on childhood obesity by adding their cross-product term into the multivariate logistic regression model.

A two-tailed *p* value < 0.05 was considered statistically significant. All statistical analyses were performed using R software (version 4.4.2).

## Results

### Participant characteristics

Table 1 shows the general characteristics comparison between the obese (*N* = 50) and normal-weight (*N* = 50) groups. There were no significant differences in children’s gender and age between the two groups (both *p* > 0.05). However, children with obesity were more likely to be born to mothers with GDM (*p* = 0.026). Moreover, children with obesity tended to have lower serum choline levels (*p* = 0.023) but higher serum creatinine and carnitine levels (*p* < 0.001 and *p* = 0.006, respectively). No statistically significant differences were observed for serum betaine, TMA and TMAO levels between the two groups (all *p* > 0.05).

**Table 1:** General characteristics comparison between the obese and normal-weight groups (*N* = 100)

		Obese group (N = 50)	Normal-weight group (N = 50)	*P* **value**
Gender				0.309
Boys		32 (64%)	27 (54%)	
Girls		18 (36%)	23 (46%)	
Age, year		5.2±0.7	4.8±1.0	0.055
Maternal GDM status				**0.026**
No		31 (62.00)	41 (82.00)	
Yes		19 (38.00)	9 (18.00)	
Height, cm		114±7.3	107±6.7	** < 0.001**
Weight, kg		25.9±4.7	17.5±2.4	** < 0.001**
BMI, kg/m^2^		19.9±1.4	15.2±0.9	** < 0.001**
Betaine, μmol/L	mean±SD	60.1±13.9	61.1±15.4	0.742
	*M* (*P*_25_, *P*_75_)	60 (47.2, 69.1)	60.2 (51, 70.4)	
Choline, μmol/L	mean±SD	46.2±13.4	52.7±14.7	**0.023**
	*M* (*P*_25_, *P*_75_)	42 (36.7, 57.2)	49.1 (43.2, 63)	
Creatinine, μmol/L	mean±SD	44.4±6.8	37.3±6.1	** < 0.001**
	*M* (*P*_25_, *P*_75_)	44.8 (39.3, 49.7)	35.9 (33.2, 42.3)	
Carnitine, μmol/L	mean±SD	49.2±6.0	46.0±5.1	**0.006**
	*M* (*P*_25_, *P*_75_)	49 (44.2, 54.5)	46 (42.9, 48.6)	
TMA, μmol/L	mean±SD	0.1±0.1	0.1±0.1	0.680
	*M* (*P*_25_, *P*_75_)	0.1 (0.1, 0.1)	0.1 (0.1, 0.2)	
TMAO, μmol/L	mean±SD	3.4±2.8	3.5±4.0	0.857
	*M* (*P*_25_, *P*_75_)	2.4 (1.7, 4.5)	2.6 (1.6, 3.9)	

Data are shown as means (SD) or n (%). Significant differences in general characteristics between the two groups are highlighted in bold. Abbreviations: BMI, body mass index; GDM, gestational diabetes mellitus; TMA, Trimethylamine; TMAO, Trimethylamine N-Oxide

## Associations of TMAO and its precursors with childhood obesity

Several associations were identified in both unadjusted and adjusted models (*P*-trend < 0.05) (Table 2). Regarding the association between choline levels and childhood obesity, after adjusted for gender, age and maternal GDM status, the OR (95 % CI) for quartile 2, quartile 3 and quartile 4 groups compared with the lowest quartile (i.e., quintile 1) were 0.25 (0.07, 0.92), 0.21 (0.06, 0.75) and 0.16 (0.04, 0.61), respectively (all *p <* 0.05), indicating higher choline was associated with decreased odds of childhood obesity. However, elevated creatinine and carnitine were associated with increased odds of childhood obesity (quartile 4 vs. quartile 1: 30.03 (5.74, 157.21), *p* < 0.001, and 5.31 (1.47, 19.16), *p* = 0.011, respectively). There were no significant associations between betaine, TMA or TMAO quartiles and childhood obesity (all *p* > 0.05).

**Table 2:** The association of TMAO and its precursors with childhood obesity

		Quartile 1	Quartile 2		Quartile 3		Quartile 4		*P***-trend**
			OR (95% CI)	*P* **value**	OR (95% CI)	*P* **value**	OR (95% CI)	*P* **value**	
Betaine	Range, μmol/L	31.42–50.58	50.59–60.21		60.22–69.45		69.46–93.88		
	Unadjusted model	Ref	0.62 (0.2, 1.89)	0.397	0.85 (0.28, 2.59)	0.777	0.73 (0.24, 2.21)	0.572	0.721
	Adjusted model	Ref	0.67 (0.2, 2.21)	0.509	1.03 (0.31, 3.43)	0.961	1 (0.29, 3.43)	0.998	0.807
Choline	Range, μmol/L	22.07–39.30	39.31–47.07		47.08–59.55		59.56–100.96		
	Unadjusted model	Ref	0.36 (0.11, 1.16)	0.087	0.26 (0.08, 0.85)	**0.025**	0.26 (0.08, 0.85)	**0.025**	**0.022**
	Adjusted model	Ref	0.25 (0.07, 0.92)	**0.037**	0.21 (0.06, 0.75)	**0.016**	0.16 (0.04, 0.61)	**0.007**	**0.008**
Creatinine	Range, μmol/L	25.00–34.80	34.81–40.68		40.69–46.26		46.27–60.01		
	Unadjusted model	Ref	3.14 (0.89, 11.06)	0.075	3.69 (1.05, 12.96)	**0.041**	29.33 (6.2, 138.78)	** < 0.001**	** < 0.001**
	Adjusted model	Ref	3.18 (0.84, 12.07)	0.089	3.25 (0.86, 12.24)	0.081	30.03 (5.74, 157.21)	** < 0.001**	** < 0.001**
Carnitine	Range, μmol/L	33.10–43.71	43.72–46.80		46.81–51.28		51.29–60.94		
	Unadjusted model	Ref	0.71 (0.22, 2.25)	0.556	1.91 (0.62, 5.88)	0.26	3.86 (1.18, 12.61)	**0.025**	**0.009**
	Adjusted model	Ref	0.93 (0.27, 3.17)	0.901	2.1 (0.63, 7.05)	0.228	5.31 (1.47, 19.16)	**0.011**	**0.005**
TMA	Range, μmol/L	0.03–0.08	0.09–0.11		0.12–0.15		0.16–0.32		
	Unadjusted model	Ref	1 (0.33, 3.06)	1	2.26 (0.73, 7.05)	0.159	1.17 (0.39, 3.58)	0.777	0.475
	Adjusted model	Ref	0.88 (0.27, 2.86)	0.829	1.99 (0.6, 6.63)	0.265	0.92 (0.28, 3.03)	0.885	0.766
TMAO	Range, μmol/L	0.47–1.66	1.67–2.51		2.52–4.00		4.01–27.5		
	Unadjusted model	Ref	1.38 (0.45, 4.2)	0.572	0.61 (0.2, 1.89)	0.391	1.62 (0.53, 4.98)	0.396	0.721
	Adjusted model	Ref	2.12 (0.59, 7.54)	0.247	0.64 (0.19, 2.15)	0.473	2.14 (0.64, 7.15)	0.217	0.584

Significant associations are highlighted in bold. Adjusted model: adjusted for gender, age and maternal GDM status. Abbreviations: CI, confidence interval; OR, odds ratios; TMA, Trimethylamine; TMAO, Trimethylamine N-Oxide

We also performed a sensitivity analysis by reclassifying the nutritional status of children in our study using the WHO Child Growth Standards. The results were generally consistent with the above results drawn from the national standard of China (Supplementary Table 1). That is, higher serum choline levels were associated with lower odds of childhood obesity, while elevated serum carnitine and creatinine levels were linked to higher odds.

**Fig. 1:**
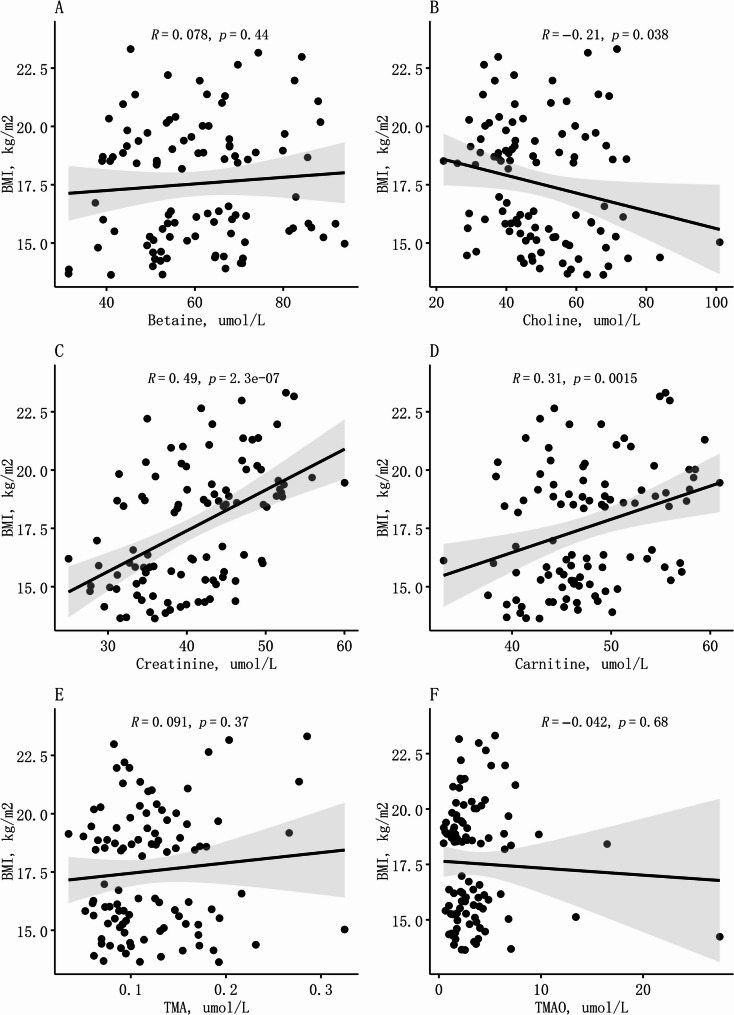
Scatterplot of TMAO and its precursors associated with BMI. The associations of TMAO and its precursors with BMI were analyzed by Pearson correlation analyses. Abbreviations: CI, confidence interval; GDM, gestational diabetes mellitus; OR, odds ratios; TMA, Trimethylamine; TMAO, trimethylamine N-Oxide

Additionally, we examined the associations of TMAO and its precursors with BMI, and found consistent results (Fig. 1). Serum choline was negatively correlated with BMI (*R* = −0.21, *p* = 0.038), whereas serum creatinine and carnitine were positively correlated with BMI (*R* = 0.49, *p* < 0.001 and *R* = 0.31, *p* = 0.002, respectively).

## Subgroup and interaction analysis

**Fig. 2:**
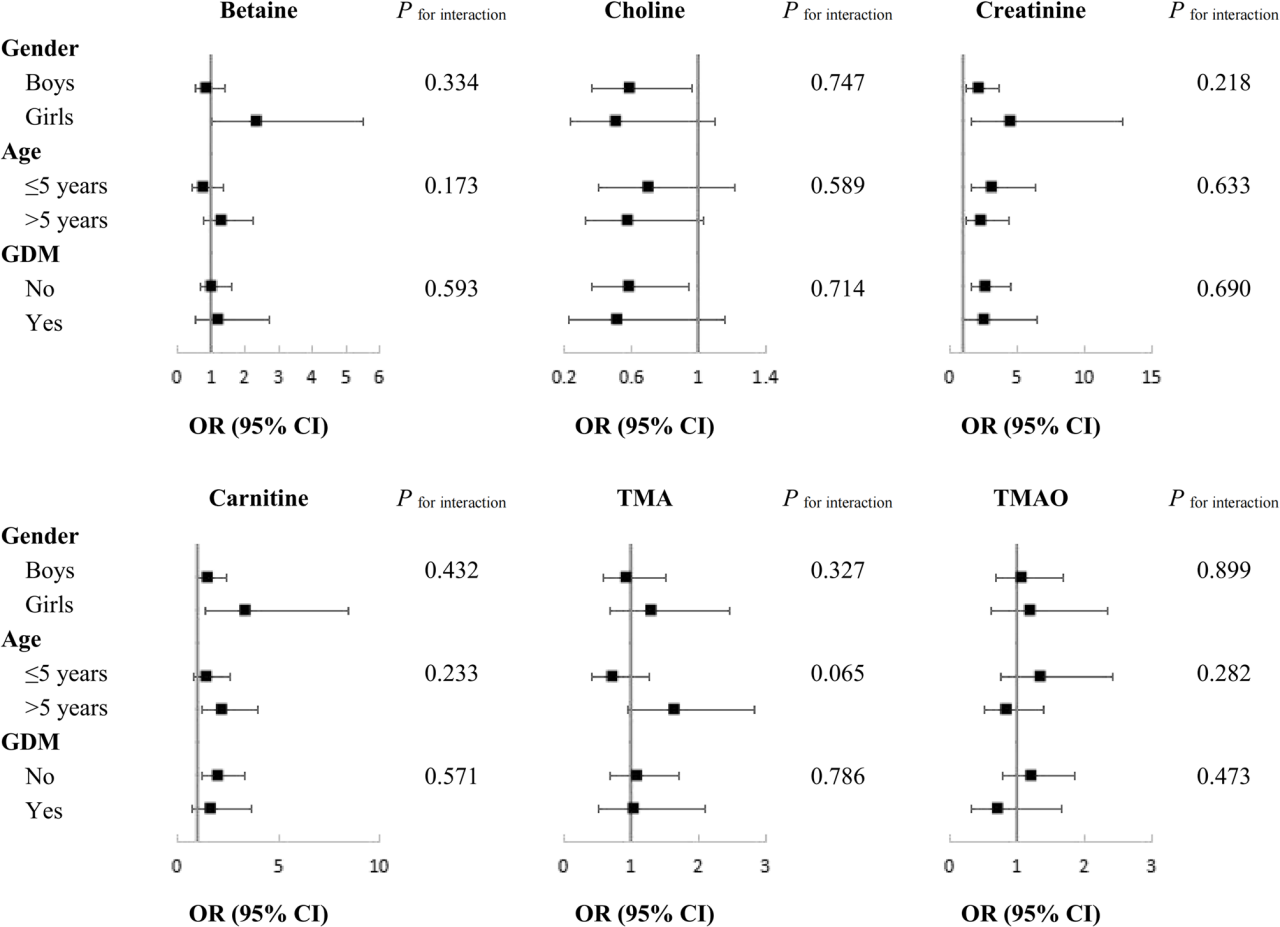
The association of TMAO and its precursors with childhood obesity stratified by baseline characteristics. Multivariate-adjusted ORs and 95% CIs of TMAO and its precursors with childhood obesity were estimated from logistic regression models. Abbreviations: CI, confidence interval; GDM, gestational diabetes mellitus; OR, odds ratios; TMA, Trimethylamine; TMAO, trimethylamine N-Oxide

We also conducted stratified analyses according to the basic clinical characteristics, including gender, age groups, and maternal GDM status, to evaluate whether these factors could modify the association of TMAO and its precursors with childhood obesity. Serum choline was consistently inversely associated with childhood obesity in boys and girls, as well as other subgroups (all OR < 1, Fig. 2). Creatinine and carnitine were consistently positively associated with childhood obesity in the subgroups (all OR > 1), although the associations in some subgroups were not significant, which might be due to reduced sample size and statistic power. Similar to the primary results, no significant associations of betaine, TMA and TMAO with childhood obesity were detected in the subgroups (all *p* > 0.05). There were also no significant interactions between TMAO-related metabolites and the basic clinical factors (all *P*_for interaciton_ > 0.05).

## Discussion

In this case-control study, we explored the associations of TMAO and its precursors with childhood obesity in Shenzhen, China. Our findings revealed several key insights into the potential role of these gut microbiota-derived metabolites in obesity development. Specifically, higher serum choline levels were associated with lower odds of childhood obesity, while elevated serum carnitine and creatinine levels were linked to higher odds.

## Comparison with previous studies

Choline, an essential nutrient, plays a crucial role in neurotransmitter synthesis, cell-membrane signaling, lipid transport, and methyl-group metabolism [19, 20]. Our findings suggest that higher serum choline levels were associated with decreased odds of childhood obesity, aligning with the study by Chang et al. [21], which identified a link between higher dietary choline and betaine intake and decreased visceral obesity-related hepatic steatosis. Additionally, Edwards et al. [22] reported that increased choline intake is associated with more efficient neural processing among overweight and obese adults during attention tasks, further supporting the role of choline in obesity and metabolic health.

The inverse relationship between choline and obesity may involve multiple mechanisms. Firstly, as a precursor for phosphatidylcholine, choline is essential for maintaining cellular membrane integrity and function. Decreased choline might result in alterations in membrane phospholipid composition, therefore impacting cellular function and energy metabolism, and leading to obesity [23]. Secondly, choline’s role in methylation pathways is crucial for regulating gene expression and controlling fat synthesis [24, 25]. Adequate choline intake may influence methylation status, thereby affecting gene expression related to obesity [25]. Furthermore, choline is vital for the synthesis of the neurotransmitter acetylcholine [20], which plays a role in appetite regulation and energy balance [26]. Sufficient choline intake may help maintain normal appetite control and energy metabolism, reducing the risk of obesity and related complications. However, the relationship between choline and obesity is likely influenced by various factors, including dietary habits, lifestyle, and genetic factors.

The positive associations between serum carnitine with childhood obesity are also noteworthy. However, with a striking effect size (OR = 5.31), its interpretation requires considerable caution. Carnitine has well-established and important roles in human muscle bioenergetics, involving the transport of long-chain fatty acids into the mitochondria for β-oxidation, thereby promoting energy expenditure [27, 28]. Traditionally, higher levels of L-carnitine have been perceived as beneficial for weight management and metabolic health. Recent systematic reviews and meta-analysis highlighted that L-carnitine supplementation significantly reduced body weight and BMI, especially in overweight and obese adults [29–31]. However, its effects on children remain less explored. In addition, some studies suggests that elevated serum carnitine levels may correlate with obesity-related metabolic diseases. For instance, a study by Koeth et al. [32] highlighted that increased dietary carnitine intake, particularly from red meat, accelerates atherosclerosis. This finding raises concerns about the metabolic consequences of elevated carnitine levels, suggesting that rather than promoting fat oxidation, high levels may contribute to metabolic dysregulation.

One potential mechanism for the observed association between high serum carnitine levels and increased odds of obesity in children is the alteration of gut microbiota composition. It was demonstrated in mice that chronic dietary L-carnitine supplementation altered cecal microbial composition, with an enrichment for taxa better suited for synthesis of TMA and TMAO [32]. Gut dysbiosis may impair the ability of gut to regulate energy balance and fat metabolism through short-chain fatty acids (SCFAs), bacterial metabolite trimethylamine, and metabolism of bile acids, thereby disrupting host metabolism and contributing to the development of obesity [33]. Moreover, the association between carnitine and obesity may also involve pathways of oxidative stress (e.g., inhibition of NF-κB expression) and chronic low-grade inflammation (e.g., decreased levels of IL-6, TNF-α, and CRP), which are hallmarks of obesity [34]. However, the striking effect size and variability should be interpreted as a call for further investigation—including longitudinal studies and research into potential confounding factors—rather than as definitive evidence of a causal role. The biological implications of this association, and whether carnitine acts as a marker, mediator, or consequence of childhood obesity, remain to be elucidated.

Creatinine, a byproduct of muscle metabolism, is conventionally regarded as a marker of renal function. Aligns with our findings, a significant body of literature supports the link between serum creatinine, childhood obesity and renal function, indicating that elevated creatinine levels may be an early indicator of obesity-related metabolic diseases [35–37].

The mechanistic underpinnings of this association likely involve several pathways. One such pathway is the renin-angiotensin-aldosterone system (RAAS), which is often activated in states of obesity, leading to increased renal blood flow and filtration rate, and consequently, higher creatinine clearance [38]. Additionally, inflammation and oxidative stress, which are commonly increased in obese individuals, can impair renal function and influence creatinine levels, potentially exacerbating the risk of obesity [39].

In the current study, we observed no significant association between serum TMAO levels and pediatric obesity, which contrasts with previous researches suggesting a link between TMAO and obesity in adults [8, 9]. The discrepancy may due to several factors. Firstly, the metabolic pathways and gut microbiota composition differ significantly between childhood and adulthood, potentially influencing TMAO production and metabolism [14, 15]. Children have a distinct gut microbiome that matures with age, which might modulate TMAO levels differently than in adults. Secondly, dietary intake and physical activity levels, which are known to impact TMAO concentrations, may vary greatly between children and adult populations, leading to different associations with obesity. Additionally, the cross-sectional nature of our study limits the ability to establish causality, unlike some longitudinal studies in adults that have observed such associations [40–42]. Lastly, the relatively small sample size of our study may have reduced the power to detect significant associations.

Notably, our null finding also contrasts with a recent pediatric study by Mihuta et al. [7], which detected a positive association between TMAO and obesity in children aged 4–18 years. Population differences might explain this inconsistency. Our study focused exclusively on early childhood (2–6 years), a period of dynamic development in gut microbiota and metabolism, whereas the participants of Mihuta et al. [7] included older children and adolescents. Moreover, differences in dietary patterns between the Romanian [7] and our Chinese population may also lead to variations in TMAO production and its metabolic impact, contributing to the divergent results.

Our subgroup analyses revealed generally consistent associations of serum choline, carnitine and creatinine, with childhood obesity across different subgroups, suggesting that these relationships may be generalizable. However, the lack of significant interactions between TMAO-related metabolites and basic clinical factors suggests that the associations are not significantly modified by these factors. This could be due to the limited sample size or the complexity of the interactions between TMAO-related metabolites and other factors.

## Clinical implication and public health significance

Our study, along with previous literature, highlights these gut microbiota-derived metabolites as potential biomarkers or factors related to childhood obesity. Our results suggest that higher choline levels were associated with lower odds of childhood obesity, which could guide dietary recommendations for obese or at-risk children, potentially improving metabolic health outcomes. The positive association between serum carnitine and obesity challenges traditional views on its role in weight management, indicating a need for caution in L-carnitine supplementation, especially in children. Elevated creatinine levels may signal early metabolic complications in children with obesity, underscoring the importance of renal function assessment in pediatric obesity management.

From a public health perspective, these findings emphasize again the importance of early intervention and prevention strategies for childhood obesity. In addition, the potential link between gut microbiota-derived metabolites and obesity suggests that modulating gut health could be a novel approach to obesity prevention. Further research is essential to apply these findings into actionable public health strategies, ultimately aiming to reduce the burden of obesity and associated metabolic disorders in children.

## Strengths and limitations

Our study provides important evidence for exploring the association of TMAO and its precursors with obesity in the young children aged 2 to 6. Additionally, our study benefits from a well-matched population and the use of a standardized UHPLC-MS/MS technique for quantifying TMAO and its precursors.

However, our study also has several limitations. The relatively small sample size may limit the power to detect significant associations, particularly in subgroup analyses. Secondly, the cross-sectional design of our study precludes causal inferences. Furthermore, other obesity-related variables such as waist and hip circumferences, body fat distribution and composition, were not measured, impeding us to further explore the link between TMAO and its precursors and childhood obesity. In addition, unmeasured confounding factors such as diet and physical activity factors may also affect the relationship between TMAO and its precursors and childhood obesity.

## Conclusion

In conclusion, our study provides evidence for the associations of TMAO and its precursors with childhood obesity. While serum choline appears to be inversely associated with childhood obesity, serum carnitine and creatinine show positive associations, shedding some new light on the potential clinical implication of these gut microbiota-derived metabolites in the management and prevention of childhood obesity. More studies are warranted to confirm our findings and further explore the associations between gut microbiota-derived metabolites and childhood obesity.

## Electronic supplementary material

Below is the link to the electronic supplementary material.

Supplementary Material 1

Supplementary Material 2

## Data Availability

Data for this study is available upon request.

## Abbreviations

BMI: Body mass index
CI: Confidence interval
FMO3: Flavin-containing monooxygenase-3
GDM: Gestational diabetes mellitus
OR: Odds ratios
RAAS: Renin-angiotensin-aldosterone system
SD: Standard deviation
SCFAs: Short-chain fatty acids
TMA: Trimethylamine
TMAO: Trimethylamine N-Oxide
UHPLC-MS/MS: Ultra high performance liquid chromatography coupled tandem mass spectrometry
