# Supplementary material for: Associations of trimethylamine N-oxide (TMAO) and its precursors with childhood obesity: a case-control study
Source: BMC Endocr Disord. 2025 Nov 25;25:273. doi: 10.1186/s12902-025-02075-z (PMC12645746; doi:10.1186/s12902-025-02075-z)
Supplement: Supplementary file 1 — Supplementary Material 1 [file 12902_2025_2075_MOESM1_ESM.docx]

**Supplementary Methods**

**Calibration and quality control (QC)**

Quantification was achieved using a multi-point calibration curve. The calibration standards ranged from 0 (lower limit of quantification, LLOQ) to 20,000 ng/mL (upper limit of quantification, ULOQ) for choline, betaine, carnitine, creatinine, and TMAO. For TMA, the calibration range was 0 (LLOQ) to 80 ng/mL (ULOQ). An 11-point calibration curve (including zero) was used for the former analytes, and a 6-point curve was used for TMA (Table 1). All calibration curves exhibited excellent linearity (r² > 0.99) and were fitted using a weighted (1/x) linear regression model to minimize heteroscedasticity.

Table 1. The calibration for all analytes.

| Sample Name | Sample Type | Choline | Betaine | Carnitine | Creatinine | TMAO | TMA |
| --- | --- | --- | --- | --- | --- | --- | --- |
| s0 | Standard | 0 | 0 | 0 | 0 | 0 | 0 |
| s1 | Standard | 40.19 | 40.19 | 40.19 | 40.19 | 40.19 | 5 |
| s2 | Standard | 80.38 | 80.38 | 80.38 | 80.38 | 80.38 | 10 |
| s3 | Standard | 160.75 | 160.75 | 160.75 | 160.75 | 160.75 | 20 |
| s4 | Standard | 312.5 | 312.5 | 312.5 | 312.5 | 312.5 | 40 |
| s5 | Standard | 625 | 625 | 625 | 625 | 625 | 80 |
| s6 | Standard | 1250 | 1250 | 1250 | 1250 | 1250 | N/A |
| s7 | Standard | 2500 | 2500 | 2500 | 2500 | 2500 | N/A |
| s8 | Standard | 5000 | 5000 | 5000 | 5000 | 5000 | N/A |
| s9 | Standard | 10000 | 10000 | 10000 | 10000 | 10000 | N/A |
| s10 | Standard | 20000 | 20000 | 20000 | 20000 | 20000 | N/A |

The precision and accuracy of the method were rigorously validated using using three replicates of QC samples. The intra-assay precision, calculated as the coefficient of variation (CV), was below 8% for all analytes (Table 2). The accuracy, expressed as the percentage deviation from the nominal concentration, was within ±15% for all QC levels. These results confirmed that the assay was highly reproducible and accurate throughout the sample analysis.

Table 2. The intra-assay precision for all analytes.

|  | Choline | Betaine | Carnitine | Creatinine | TMAO | TMA |
| --- | --- | --- | --- | --- | --- | --- |
| QC1 | 1276 | 1507 | 1411 | 1265 | 1266 | 0.1744 |
| QC2 | 1240 | 1537 | 1372 | 1255 | 1246 | 0.1877 |
| QC3 | 1260 | 1341 | 1278 | 1269 | 1271 | 0.1658 |
| Average | 1258.67 | 1461.67 | 1353.67 | 1263 | 1261 | 0.1760 |
| SD | 18.04 | 105.57 | 68.37 | 7.21 | 13.23 | 0.0110 |
| CV | 1.43% | 7.22% | 5.05% | 0.57% | 1.05% | 6.27% |

**UHPLC-MS/MS Parameters**

Serum concentrations of TMAO-related metabolites (i.e., betaine, choline, creatinine, free carnitine, TMA, and TMAO in the present study) were determined by the stable isotope dilution ultra high performance liquid chromatography coupled tandem mass spectrometry (UHPLC-MS/MS) method (QTRAP 5500, AB Sciex, MA, USA). Analysis was performed in positive electrospray ionization (ESI+) mode using multiple reaction monitoring (MRM). The Optimized MRM parameters for target analytes and internal standards were present in the Table 3.

The key mass spectrometric (MS) source parameters were optimized as follows: spray voltage was +5500V, the resolution was unit resolution, the curtain gas flow rate was 30 (arbitrary units, similarly hereinafter), the collision gas is 8 , the auxiliary gas flow rate was 55 and the heating gas velocity is 50. The ion source temperature is 500 degrees.

Table 3. Optimized MRM parameters for target analytes and internal standards

| Analyte/IS | Retention time (RT) | Precursor ion (m/z) | Product ion (m/z) | Declustering Potential (DP, V) | Collision Energy (CE, eV) |
| --- | --- | --- | --- | --- | --- |
| TMAO | 2.71 | 76.3 | 58.2 | 65 | 24 |
| Choline | 1.91 | 104.2 | 60.1 | 70 | 24 |
| Betaine | 1.74 | 118.2 | 58.2 | 70 | 36 |
| Carnitine | 2.46 | 162.1 | 103.2 | 75 | 24 |
| Creatinine | 1.27 | 114.1 | 44.1 | 100 | 26 |
| TMA | 1.66 | 99 | 58.1 | 75 | 33 |
| d9-TMAO | 2.71 | 85.2 | 66 | 70 | 27 |
| d4-choline | 1.91 | 108.2 | 60.2 | 75 | 23 |
| d9-betaine | 1.74 | 127.2 | 66.2 | 80 | 38 |
| d3-carnitine | 2.46 | 165.1 | 60.3 | 75 | 24 |
| d3-creatinine | 1.27 | 117 | 47.1 | 70 | 17 |

*Note: external standard was applied for TMA.

**Analytical Methodology Validation**

The performance of the UHPLC-MS/MS assay was monitored using QC samples. The intra-assay precision, calculated from replicate QC injections and expressed as the CV, was ≤ 7.3% for all target metabolites, demonstrating excellent analytical reproducibility during the sample analysis. In addition, the method was validated in accordance with industry standards prior to its implementation (Reference 1-5).

Reference:

[1]. Westerhuis JA, van Velzen EJ, Hoefsloot HC, Smilde AK. Multivariate paired data analysis: multilevel PLSDA versus OPLSDA. Metabolomics. 2010 Mar;6(1):119-128. doi: 10.1007/s11306-009-0185-z. Epub 2009 Oct 28. PMID: 20339442; PMCID: PMC2834771.

[2]. Thévenot EA, Roux A, Xu Y, Ezan E, Junot C. Analysis of the Human Adult Urinary Metabolome Variations with Age, Body Mass Index, and Gender by Implementing a Comprehensive Workflow for Univariate and OPLS Statistical Analyses. J Proteome Res. 2015 Aug 7;14(8):3322-35. doi: 10.1021/acs.jproteome.5b00354. Epub 2015 Jul 2. PMID: 26088811.

[3]. Goodacre R, Vaidyanathan S, Dunn WB, Harrigan GG, Kell DB. Metabolomics by numbers: acquiring and understanding global metabolite data. Trends Biotechnol. 2004 May;22(5):245-52. doi: 10.1016/j.tibtech.2004.03.007. PMID: 15109811.

[4]. Wang C, Kong H, Guan Y, Yang J, Gu J, Yang S, Xu G. Plasma phospholipid metabolic profiling and biomarkers of type 2 diabetes mellitus based on high-performance liquid chromatography/electrospray mass spectrometry and multivariate statistical analysis. Anal Chem. 2005 Jul 1;77(13):4108-16. doi: 10.1021/ac0481001. PMID: 15987116.

[5]. Want EJ, Wilson ID, Gika H, Theodoridis G, Plumb RS, Shockcor J, Holmes E, Nicholson JK. Global metabolic profiling procedures for urine using UPLC-MS. Nat Protoc. 2010 Jun;5(6):1005-18. doi: 10.1038/nprot.2010.50. PMID: 20448546.
