# Supplementary material for: Associations of trimethylamine N-oxide (TMAO) and its precursors with childhood obesity: a case-control study
Source: BMC Endocr Disord. 2025 Nov 25;25:273. doi: 10.1186/s12902-025-02075-z (PMC12645746; doi:10.1186/s12902-025-02075-z)
Supplement: Supplementary file 2 — Supplementary Material 2 [file 12902_2025_2075_MOESM2_ESM.docx]

**Supplementary Table 1. The association of TMAO and its precursors with childhood obesity defined according to the WHO Child Growth Standards.**

|  |  | **Q1** | **Q2** | |  | **Q3** | |  | **Q4** | | P-trend |
| --- | --- | --- | --- | --- | --- | --- | --- | --- | --- | --- | --- |
|  |  |  | **OR (95% CI)** | P value |  | **OR (95% CI)** | P value |  | **OR (95% CI)** | P value |  |
| Betaine | Range | 31.42-50.97 | 50.98-59.21 |  |  | 59.22-69.73 |  |  | 69.74-93.88 |  |  |
|  | Unadjusted model | Ref | 0.73 (0.21, 2.56) | 0.627 |  | 0.69 (0.18, 2.62) | 0.583 |  | 0.85 (0.24, 3) | 0.796 | 0.806 |
|  | Adjusted model | Ref | 1.12 (0.27, 4.68) | 0.88 |  | 1.2 (0.24, 6.15) | 0.824 |  | 1.76 (0.36, 8.63) | 0.487 | 0.476 |
| Choline | Range | 28.71-40.53 | 40.54-47.48 |  |  | 47.49-59.86 |  |  | 59.87-100.96 |  |  |
|  | Unadjusted model | Ref | 0.5 (0.14, 1.83) | 0.294 |  | 0.26 (0.06, 1.07) | 0.061 |  | 0.36 (0.1, 1.38) | 0.137 | 0.103 |
|  | Adjusted model | Ref | 0.31 (0.07, 1.45) | 0.139 |  | 0.16 (0.03, 0.83) | **0.029** |  | 0.2 (0.04, 0.94) | **0.041** | **0.038** |
| Creatinine | Range | 25.00-34.67 | 34.68-39.93 |  |  | 39.94-44.96 |  |  | 44.97-55.90 |  |  |
|  | Unadjusted model | Ref | 4.67 (0.85, 25.75) | 0.077 |  | 4.62 (0.81, 26.45) | 0.086 |  | 50 (7.4, 337.76) | **<0.001** | **<0.001** |
|  | Adjusted model | Ref | 4.56 (0.71, 29.4) | 0.11 |  | 2.97 (0.44, 20.1) | 0.264 |  | 41.72 (5.23, 332.61) | **<0.001** | **<0.001** |
| Carnitine | Range | 33.10-43.47 | 43.48-46.03 |  |  | 46.04-50.11 |  |  | 50.12-59.44 |  |  |
|  | Unadjusted model | Ref | 0.81 (0.22, 2.92) | 0.744 |  | 1.25 (0.33, 4.73) | 0.746 |  | 3.59 (0.98, 13.16) | 0.054 | **0.045** |
|  | Adjusted model | Ref | 1.29 (0.31, 5.37) | 0.727 |  | 1.25 (0.28, 5.65) | 0.774 |  | 6.13 (1.3, 28.91) | **0.022** | **0.032** |
| TMA | Range | 0.05-0.09 | 0.10-0.11 |  |  | 0.12-0.15 |  |  | 0.16-0.32 |  |  |
|  | Unadjusted model | Ref | 1.2 (0.3, 4.86) | 0.799 |  | 3.08 (0.81, 11.67) | 0.098 |  | 1.72 (0.45, 6.63) | 0.429 | 0.24 |
|  | Adjusted model | Ref | 0.94 (0.2, 4.4) | 0.934 |  | 2.66 (0.61, 11.65) | 0.194 |  | 1.18 (0.25, 5.44) | 0.836 | 0.497 |
| TMAO | Range | 0.48-1.68 | 1.69-2.43 |  |  | 2.44-3.97 |  |  | 3.98-27.5 |  |  |
|  | Unadjusted model | Ref | 1.86 (0.54, 6.43) | 0.329 |  | 0.46 (0.11, 1.94) | 0.293 |  | 1.35 (0.37, 4.92) | 0.649 | 0.822 |
|  | Adjusted model | Ref | 6.17 (1.23, 31.07) | **0.027** |  | 0.53 (0.1, 2.69) | 0.441 |  | 2.89 (0.62, 13.4) | 0.176 | 0.832 |

Significant associations are highlighted in bold. Adjusted model: adjusted for gender, age and maternal gestational diabetes mellitus (GDM) status.

Abbreviations: CI, confidence interval; OR, odds ratios; TMA, Trimethylamine ; TMAO, Trimethylamine N-Oxide.
